# Osteoprotegerin Polymorphisms in a Mexican Population with Rheumatoid Arthritis and Generalized Osteoporosis: A Preliminary Report

**DOI:** 10.1155/2015/376197

**Published:** 2015-05-03

**Authors:** Maria Guadalupe Zavala-Cerna, Maria Cristina Moran-Moguel, Jesus Alejandro Cornejo-Toledo, Norma Guadalupe Gonzalez-Montoya, Jose Sanchez-Corona, Mario Salazar-Paramo, Arnulfo Hernan Nava-Zavala, Erika Anita Aguilar-Chavez, Miriam Fabiola Alcaraz-Lopez, Alicia Guadalupe Gonzalez-Sanchez, Laura Gonzalez-Lopez, Jorge Ivan Gamez-Nava

**Affiliations:** ^1^Programa Internacional, Facultad de Medicina, Universidad Autonoma de Guadalajara, 44100 Zapopan, JAL, Mexico; ^2^Division de Medicina Molecular del Centro de Investigacion Biomedica de Occidente, Instituto Mexicano del Seguro Social, 44340 Guadalajara, JAL, Mexico; ^3^Programa de Becarios en Investigacion del Instituto Mexicano del Seguro Social, Programa de Doctorado en Farmacologia, Centro Universitario de Ciencias de la Salud, Universidad de Guadalajara, 44340 Guadalajara, JAL, Mexico; ^4^Division de Investigacion en Salud, UMAE, Hospital de Especialidades, CMNO, IMSS and Departamento de Fisiologia, CUCS, Universidad de Guadalajara, 44340 Guadalajara, JAL, Mexico; ^5^Unidad de Investigacion en Epidemiologia Clinica, Hospital de Especialidades, Centro Medico Nacional de Occidente del Instituto Mexicano del Seguro Social, 44340 Guadalajara, JAL, Mexico; ^6^Servicio de Medicina Interna, Inmunologia y Reumatologia, Hospital General de Occidente, Secretaria de Salud Jalisco, 45170 Guadalajara, JAL, Mexico; ^7^Unidad de Medicina Familiar 2, Instituto Mexicano del Seguro Social, 44340 Guadalajara, JAL, Mexico; ^8^Departamento de Medicina Interna-Reumatologia, Hospital General de Zona 14, Instituto Mexicano del Seguro Social, 44860 Guadalajara, JAL, Mexico; ^9^Departamento de Medicina Interna-Reumatologia del Hospital General Regional No. 110, Instituto Mexicano del Seguro Social y Programa de Doctorado en Salud Publica, CUCS, Universidad de Guadalajara, 44340 Guadalajara, JAL, Mexico; ^10^Unidad de Investigación en Epidemiologia Clinica, Hospital de Especialidades, Centro Medico Nacional de Occidente del Instituto Mexicano del Seguro Social y Programa de Doctorado en Salud Publica, CUCS, Universidad de Guadalajara, 44340 Guadalajara, JAL, Mexico

## Abstract

Bone disease in rheumatoid arthritis (RA) is a complex phenomenon where genetic risk factors have been partially evaluated. The system formed by receptor activator for nuclear factor-*κ*B (RANK), receptor activator for nuclear factor-*κ*B ligand (RANKL), and osteoprotegerin (OPG): RANK/RANKL/OPG is a crucial molecular pathway for coupling between osteoblasts and osteoclasts, since OPG is able to inhibit osteoclast differentiation and activation. We aim to evaluate the association between SNPs C950T (rs2073617), C209T (rs3134069), T245G (rs3134070) in the *TNFRSF11B* (OPG) gene, and osteoporosis in RA. We included 81 women with RA and 52 healthy subjects in a cross-sectional study, genotyped them, and measured bone mineral density (BMD) at the lumbar spine and the femoral neck. Mean age in RA was 50 ± 12 with disease duration of 12 ± 8 years. According to BMD results, 23 (33.3%) were normal and 46 (66.7%) had osteopenia/osteoporosis. We found a higher prevalence of C allele for C950T SNP in RA. Polymorphisms C209T and T245G did not reach statistical significance in allele distribution. Further studies including patients from other regions of Latin America with a multicenter design to increase the sample size are required to confirm our findings and elucidate if C950T SNP could be associated with osteoporosis in RA.

## 1. Introduction

Rheumatoid arthritis (RA) is a chronic inflammatory disease that affects synovial joints [1]. Its prevalence in Mexico is considerably elevated ranging from 0.3 to 1% [2]. Currently, the diagnosis of osteoporosis is made on the basis of a measurement by dual-energy x-ray absorptiometry (DXA) at the spine and hip showing a decrement in bone mineral density (BMD) lower than 2.5 standard deviations in comparison with healthy young adults [3]. However, bone involvement in RA is complex; an increased proportion of patients may suffer from osteoporosis; nevertheless causal effects are considered to be multifactorial. Previous studies performed in our center showed a prevalence of axial osteoporosis in Mexican women with RA of 31% [4].

Genetic risk factors constitute a variable that contributes to influencing the development of osteoporosis in RA, in this context; some genetic polymorphisms may influence the rate of presentation of osteoporosis. Nevertheless such genetic changes do not explain entirely osteoporosis presence or fracture related events. Strong evidence supports the fact that osteoclasts have an important role in osteoporosis and bone erosion formation [5, 6].

The system conformed by nuclear factor-*κ*B (RANK)/receptor activator for nuclear factor-*κ*B ligand (RANKL) and osteoprotegerin (OPG) are crucial, for the bone remodeling process. Furthermore, it appeared that not only osteoblasts but also activated *T* lymphocytes play a crucial role in the pathogenesis of rheumatoid arthritis (RA) and osteoporosis, since they can produce RANKL, which stimulates the differentiation and activation of osteoclasts [7]. These three proteins belong to the superfamily of the TNF-*α* and have different patterns of expression; RANKL is expressed in osteoblasts and its function is to participate in osteoclasts differentiation [7, 8]. Osteoprotegerin (OPG) lacks a transmembrane domain, and hence it is a receptor that can be secreted. OPG recognizes and is able to bind RANKL, blocking its interaction with RANK inhibiting osteoclastic differentiation and activation [9–11]. Since OPG is homologous to RANK it acts as a false receptor for RANKL [12–14]. OPG is secreted mainly by stromal cells but can also be secreted by other types of cells [15]. Different studies have been able to identify single nucleotide polymorphisms (SNPs) in* TNFSF11* (RANKL) and* TNFRSF11B* (OPG) genes in association with osteoporosis or low bone density [12, 16]. It has been documented that OPG overexpression induces an increase in bone density and protects from the development to osteoporosis. In contrast mice that are OPG-deficient develop osteoporosis very rapidly [14, 15, 17]. OPG overexpression blocks osteoclasts maturation; contrariwise, an underexpression of this molecule results in an excessive increase of bone resorption and osteoporosis [18]. Mutations in the promoter region of* TNFRSF11B* could have influence on the transcription and translation rate of OPG. A study in European population (Eslovenia) showed that two SNPs named rs3134069 and rs3134070 in the* TNFRSF11B* could form a haplotype with susceptibility to osteoporosis [16, 19]. To date, there is a lack of information evaluating the relationship between polymorphisms of OPG gene and bone mineral density.

Therefore, the aim of the present study was to assess if there is an association between SNPs C950T (rs2073617), C2097 (rs3134069), and T245G (rs3134070) in the* TNFRSF11B* (OPG) gene and osteoporosis in rheumatoid arthritis patients.

## 2. Patients and Methods

### 2.1. Study Subjects

We carried out a cross-sectional study that included 81 consecutive unrelated women who were diagnosed with RA from March 2007 to March 2009. These patients were referred from an outpatient rheumatology clinic in a secondary care center in Guadalajara, Mexico (Hospital General Regional 110, IMSS). Inclusion criteria were as follows: age ≥ 18 years at entry; self-identified race Mexican Mestizo (defined as individuals who were born in Mexico and were descendants of the original autochthonous inhabitants of the region and of individuals mainly Spaniards); fulfillment of the 1987 American College of Rheumatology (ACR) classification criteria [20]; and no previous BMD measurement. Patients were excluded if pregnancy was present and if they had an overlap syndrome, were receiving bisphosphonates or parathyroid hormone therapy, or had a comorbidity associated with low BMD, such as diabetes mellitus, thyroid disease, or chronic renal failure; patients were not related to each other.

A healthy control group matched by age and sex was obtained from blood donors who attended to “Centro Médico Nacional de Occidente” blood bank; all blood donors were of Mexican Mestizo origin and denied at the time of the study having any disease (acute or chronic).

### 2.2. Clinical Assessment

Each patient was interviewed using a structured questionnaire to record demographic information, general risk factors for osteoporosis (i.e., age, weight, and height), clinical characteristics, and RA treatment. At the time of the evaluation two trained researchers assessed RA disease activity by systematical evaluation of the DAS-28 index [21]; to determine patient disability the Spanish modified version of the Health Assessment Questionnaire-Disability Index (HAQ-DI) [22] was used. Global functional status was evaluated according to the Steinbrocker classification [23]. Rheumatoid factor and C-reactive protein (CRP) were measured by nephelometry using commercially available kits. Erythrocyte sedimentation rate (ESR, mm/h) was measured using the Wintrobe method.

### 2.3. BMD Measurement

BMD was measured (g/cm^2^) by DEXA using a Lunar Prodigy densitometer (GE Medical Systems Lunar, Madison, WI, software V 8.8; GE Medical Systems); the anatomical regions assessed were lumbar spine in the posterior-anterior projection (L1-L4) and femoral neck. The coefficient of variation during the measurement of a standard phantom in our laboratory is 0.7%. The coefficient of variation was 2.4% at the lumbar spine and 1.6% at the femoral neck. All scans were performed by the same experienced technician, who was blinded to the characteristics of patients. Each patient was classified into one of the following categories proposed by WHO: normal BMD, defined as having a measurement of BMD within 1 SD of the BMD of normal young adult women (equivalent to *T* score > −1); osteopenia, defined as having a *T* score in BMD between −1 and −2.4 SD; and osteoporosis, defined as having a *T* score in BMD ≤ −2.5 SD. Patients with a *T* score < −1.0 were considered to have low BMD in consequence; for analysis purposes in our study we decided to include osteopenia and osteoporosis in one single group denominated as “low BMD”.

### 2.4. Genotyping

DNA from 81 patients with rheumatoid arthritis was extracted from blood samples using conventional methods [24] and stored frozen at −80°C.

We chose three SNPs that are present in the promoter region (5′UTR) of the* TNFRSF11B* (OPG) gene; such polymorphisms were previously reported in association with osteoporosis in postmenopausal women or related conditions [19, 25–28]. We performed 3 polymerase chain reaction-restriction fragment length polymorphisms (PCR_RFLPs) protocols as previously described [26, 29, 30].  Table 1 shows primer sequence, products obtained after PCR, restriction enzymes used, their recognition site, and end products after restriction.

Each PCR reaction was carried out in 10 *μ*L final volume containing (final concentrations) 1X buffer (200 mM Tris-HCl pH 8.4, 500 mM KCl, and 4 mM MgCl_2_); 5 pmol/mL each of the pair primers according to polymorphism (Table 1); 10 mM each of the four deoxyribonucleoside triphosphates; 1 U of taq DNA polymerase (Invitrogen, Carlsbad, CA, USA); and 200 to 300 ng DNA template. The PCR products were visualized by electrophoresis in 10% polyacrylamide gels at 150 V for 90 min, followed by silver staining.

### 2.5. Statistical Analysis

Allelic and genotypic frequencies for each SNP were determined by gene count. Hardy-Weinberg equilibrium was tested, using *χ* ^2^ test. Genotypic differences between RA patients with and without osteoporosis were evaluated by Mantel-Haenszel test using the EPI INFO (version 6.04d) statistical program. For allelic differences, we used Fisher's exact test. Odds ratios (OR) and 95% confidence interval (95% CI) were computed to evaluate the risk for osteoporosis conferred by presence of the risk alleles. All analyses used two-sided tails with a *P* value of ≤0.05 used as significance criterion.

## 3. Results

### 3.1. Demographics and Clinical

We included 81 patients, classified as RA according to ACR classification criteria [20], and 52 healthy subjects. For the RA group, mean age was 50 ± 12 years, 100% of the studied subjects were women, mean duration disease was 12 ± 8 years, and all of them were receiving treatment, some of them with monotherapy and others combined therapy; the number and percentages of patients taking a specific drug are listed in Table 2.

RA patients were evaluated for BMD according to the classification criteria of the WHO for osteoporosis. According to the results of bone densitometry 23 patients (33.3%) had normal BMD, 31 (44.9%) had osteopenia (low bone density), and 15 (21.7%) had osteoporosis. For study purposes we grouped osteopenia with osteoporosis in one group constituted by 46 patients (66.7%) (see Table 2). From these, 23 patients (32.9%) had osteopenia and 13 (18.6%) had osteoporosis in the lumbar spine, whereas 35 patients (50.7%) had osteopenia and 7 (10.1%) had osteoporosis of the femoral neck. Clinical risk factors and factors related to RA severity for low BMD were registered and are listed in Table 2.

### 3.2. Molecular Analysis

We performed at least three PCR-RFLP tests for every subject included in the present study but were able to obtain complete genotyping results in 77 DNA samples from RA patients and 52 healthy subjects. Healthy subjects group was in Hardy-Weinberg equilibrium (data not shown).

In Table 3 genotype and allele frequencies of three SNPs in the OPG gene in healthy subjects and rheumatoid arthritis patients are shown; we found significant difference in the distribution of the C950T (rs2073617) alleles, the C allele being more prevalent in the RA group. Polymorphisms C209T and T245G did not reach statistical significance.

We further analyzed genetic variants in RA patients and divided this group with respect to bone mass index status according to the WHO classification in normal or osteopenia/osteoporosis; there was no significant difference in allele distribution between RA patients with and without osteoporosis; Table 4 shows genotype and allele frequencies in these subgroups.

## 4. Discussion

Osteoporosis represents a major health problem throughout the world and the most serious consequence of osteoporosis is hip fracture, which has a high associated morbidity and mortality [31]. Systemic effects of RA in bone remodeling include loss of axial and appendicular bone mass, associated with an increase in fracture risk (9.6 per 1,000 person-years) [32]. Clinical patterns of bone mass loss include generalized osteoporosis (axial and peripheral), juxta-articular osteoporosis which is adjacent to synovial membrane, and marginal and subchondral erosions which are directly associated with inflamed synovial tissue [33]. A high proportion of patients will suffer from hip and spine fractures causing limitations in physical abilities and social capabilities. Osteoporosis presence in RA patients involves multiple factors, such as disease activity, the use of some prescribed drugs particularly DMARDs and corticosteroids, female sex, older age, and menopause [34].

The development of erosive lesions as well as generalized osteoporosis in RA results from an imbalance between bone mass formation and bone resorption process. In RA the unsteadiness between osteoblastic bone formation (mesenchymal origin) and osteoclastic bone resorption (derived from monocytes/macrophages) promotes bone resorption by osteoclasts and as a consequence loss in bone strength, which may lead to the development of osteoporosis and fractures.

Multiple interactions between bone and the immune system have been described; bone is a major target of chronic inflammation, since it increases bone resorption and results in suppressed local bone formation, causing a wide spectrum of bone involvement in RA [35]. Lymphocyte or macrophage-derived cytokines are among the most potent mediators of inflammation, involved in upregulation of osteoclasts formation, activation, and signaling pathways [36].

Osteoclasts progenitors are recruited under physiological conditions from the haematopoyetic system and can get stimulated by some cytokines and hormones to form precursors that will eventually express cell surface markers of a well-differentiated osteoclast; furthermore, some inflammatory synovial cells, present in RA, particularly mononuclear cells and giant multinucleated cells can be differentiated into osteoclasts [5], under proinflammatory cytokines stimuli, including tumor necrosis factor alpha (TNF-*α*), interleukin 1 (IL-1), and interleukin 6 (IL-6) [6].

Recently Pathak et al. described that sera from RA active patients contribute to bone loss by two mechanisms: (1) directly inhibiting osteoblasts proliferation and differentiation and (2) enhancing osteoblast-mediated osteoclastogenesis via RANKL and IL-6 [37].

Another key component that has influence over the osteoclastic differentiation is RANK, a receptor for TNF, attached to the membrane of osteoclast precursor cells that can bind to RNAKL through interaction with osteoblastic stromal cells [9, 38, 39]. RANK is essential for signal transduction that will lead to ostoclastic differentiation. Osteoprotegerin (OPG) also known as inhibition factor for osteoclastogenesis is a member of the TNFRSF, located in chromosome 8q24 [17] with a length of 29 Kb [13]; it is synthesized originally as a propeptide of 400 aa and is secreted as a soluble protein after the cleavage of 21 aa that corresponds to the transmembrane and cytoplasmic domains [17]. OPG mRNA is expressed in different human tissues (lung, heart, kidney, liver, intestines, stomach, cerebrum, thyroid gland, and bone marrow) apart from the bone, where its primary function appears to be inhibition of osteoclasts maturation and activation. In this study, we evaluated the association of three* TNFRSF11B* promoter polymorphisms with presence of osteoporosis in RA. Our study demonstrated that the C allele of the C950T was more frequent in RA compared with HS; thereafter we analyzed the RA group to seek for differences in allele distribution between patients with and without osteoporosis; nevertheless we did not observe a significant difference. The other two polymorphisms did not reach statistical significance.

The observed association in the present study could be caused by direct functional effect of the SNP or to linkage disequilibrium (LD) with another functional variant. According to the HapMap data, there is a certain amount of LD between these SNPs, indicating that although it is possible that different causative variants exist in* TNFRSF11B*, it is also possible that only one of them turns out to have functional consequences. The C950T SNP is located in the promoter region, near the TGF-*β* response area and 129 bp upstream the TATA box [40]. A functional study of* TNFRSF11B* polymorphisms through luciferase reporter assays revealed that gene expression was affected by the C950T polymorphism, since it was significantly higher in the presence of the C allele [27].

Previous association studies that include polymorphisms in the* TNFRSF11B* gene were performed in osteoporotic patients. In 2002 Ohmori et al. showed a significant association between C950T SNP and lower BMD [25]; in contrast with such results in 2004 Brändström et al. showed that C950T was not associated with bone mineral density in elderly Swedish women [41], and later Arko et al. in 2005 tested the three polymorphisms included in the present study and others searching for an association with bone mineral density in postmenopausal women; the linkage disequilibrium was confirmed but no associations were found [26]. For SNP T245G, similar frequencies as obtained in this study have been described previously by Zajíčková et al. in postmenopausal women [42]. Only a few studies have looked for an association between these SNPs and RA; in 2010 Assmann et al. performed a case-control study and found an association between SNPs in the RANK and RANKL gene but not in the OPG gene; nevertheless none of the SNPs included in our study were genotyped by them [43]. In a recent study the SNP A163G in the osteoprotegerin gene was associated with osteoporosis in RA [44]. This polymorphism was not included in the present study but a lack of association with this particular polymorphism and osteoporosis in RA was previously confirmed by our study group [45].

Because this work constitutes a preliminary report, a limitation in our study is a small sample size that may limit our statistical power to detect small differences between groups although we consider that our sample would be sufficient to identify polymorphisms that may confer a high risk for low BMD in RA and did not show any trend to suspect that increasing the sample size a statistical significance would be achieved. We recognize that future multicenter studies should be performed, increasing the sample size, with our results being useful as a referent for Mexican patients with RA to identify that these polymorphisms probably do not confer a high risk for low BMD in our population with RA. Further studies in other populations are required to confirm our findings.

In conclusion, we confirmed the association of C allele of the C950T SNP in the* TNFRSF11B* gene with RA patients, but we were not able to support our main hypothesis that this polymorphism in the OPG gene is associated with osteoporosis in RA. Further studies including other regions of Mexico and Latin America with an increased sample size are required to confirm our findings and further elucidate if C950T SNP in the OPG gene could be associated with osteoporosis and not only with RA presence.

## Figures and Tables

**Table 1:** Genotyping strategies for *TNFRSF11B* polymorphism variants detection.

SNP	Primers	Band size (bp)	Restriction enzyme	Recognized sequence	Band size after digestion (bp)
C950T rs2073617	5′-TGCGTCCGGATCTTGGCTGGATCGG-3′	548	* Hinc II *	GTY RAC	**C** 248 and 287 **T** 548
	5′-ACTTACCACGAGCGCGCAGCACAGCAA-3′				

C209T rs3134070	5′-CGAACCCTAGAGCAAAGTGC-3′	271	* Taq I *	T CGA	**C** 212, 31 and 28 **T** 212 and 59
	5′-TGTCTGATTGGCCCTAAAGC-3′				

T245G rs3134069	5′-CGAACCCTAGAGCAAAGTGC-3′	271	* Hinf I *	G ANTC	**A** 195, and 176 **C** 127, 76 and 69
	5′-TGTCTGATTGGCCCTAAAGC-3′				

SNP: single nucleotide polymorphism and bp: base pair.

**Table 2:** Clinical characteristics of study patients.

Characteristic	*n* = 81
Age (years), median (range)	49.9 (21–79)
Menopausal, *n* (%)	43 (53.1)
Weight (kg), median (range)	65.8 (43–94)
BMI, median (range)	26.87 (18–38)
Oral contraceptives use, *n* (%)	41 (50.6)
Current smoker, *n* (%)	17 (21)
Family history of fractures *n* (%)	6 (7.4)
Alcohol consumption 3 or more units/day, *n* (%)	3 (3.37)
Duration of RA (years), median (range)	11.8 (1.5–35)
DAS-28 score, median (range)	3.8 (0–6.7)
Global functional status III-IV, *n* (%)	7 (7.8)
HAQ-DI score, median (range)	0.7 (0–2.8)

Medications	*n* (%)

Glucocorticoid	72 (88.9)
Prednisone dosage (mg/day), median (range)	4.4 (0–10)
Methotrexate	56 (70.9)
Sulfasalazine	39 (49.4)
Chloroquine	27 (34.2)
Azathioprine	25 (31.6)
D-Penicillamine	10 (12.7)
Biologic agents	8 (10.1)

BMD (WHO classification)	*n* (%)

Normal	23 (33.3)
Osteopenia/osteoporosis	46 (66.7)

BMI: body mass index, RA: rheumatoid arthritis, DAS-28: disease activity score, HAQ-DI: health assessment questionnaire-disability index, BMD: bone mineral density, and WHO: World Health Organization.

**Table 3:** Genotype and allele frequencies of polymorphisms in the* TNFRSF11B *(OPG) gene in healthy subjects and rheumatoid arthritis (RA) patients.

SNP	Genotypes	HS	RA	Allele	HS	RA	*P*	OR
								(95% CI)
C950T rs2073617	CC	9 (17.6)	28 (43.1)	C	47 (46.1)	82 (63.1)	0.01	0.50 (0.30–0.85)
	CT	29 (56.9)	26 (40)	T	55 (53.9)	48 (36.9)		
	TT	13 (25.5)	11 (16.9)					

C209T rs3134070	CC	39 (75.0)	52 (72.2)	C	89 (85.6)	122 (84.7)	1.0	1.1 (0.53–2.2)
	CT	11 (21.2)	18 (25.0)	T	15 (14.4)	22 (15.3)		
	TT	2 (3.8)	2 (2.8)					

T245G rs3134069	AA	41 (78.8)	66 (85.7)	A	93 (89.4)	142 (92.2)	0.51	0.71 (0.30–1.69)
	AC	11 (21.2)	10 (13)	C	11 (10.6)	12 (7.8)		
	CC	0 (0)	1 (1.3)					

OPG: osteoprotegerin, SNP: single nucleotide polymorphism, RA: rheumatoid arthritis, HS: healthy subjects, OR: odds ratios, and 95% CI: 95% confidence intervals. In order to compute OR (95% CI) the following alleles were used as reference: in correspondence with the allele of risk: rs2073617 T, rs3134070 T, and rs3134069 C.

**Table 4:** Genotype and allele frequencies of polymorphisms in the *TNFRSF11B  *(OPG) gene in rheumatoid arthritis (RA) patients with normal and low bone mineral density (BMD).

SNP	Genotypes	RA with normal BMD	RA with low BMD	Allele	RA with normal BMD	RA with low BMD	*P *	OR
								(95% CI)
C950T rs2073617	CC	9 (50.0)	16 (44.4)	C	25 (69.4)	46 (63.9)	0.67	1.3 (0.55–3.03)
	CT	7 (38.9)	14 (38.9)	T	11 (30.6)	26 (36.1)		
	TT	2 (11.1)	6 (16.7)					

C209T rs3134070	CC	17 (81.0)	29 (69.0)	C	37 (88.1)	70 (83.3)	0.60	1.5 (0.49–4.43)
	CT	3 (14.3)	12 (28.6)	T	5 (11.9)	14 (16.7)		
	TT	1 (4.8)	1 (2.4)					

T245G rs3134069	AA	20 (90.9)	39 (88.6)	A	42 (95.5)	82 (93.2)	0.72	1.5 (0.29–7.95)
	AC	2 (9.1)	4 (9.1)	C	2 (4.5)	6 (6.8)		
	CC	0 (0)	1 (2.3)					

OPG: osteoprotegerin, SNP: single nucleotide polymorphism, RA: rheumatoid arthritis, BMD: bone mineral density, OR: odds ratios, and 95% CI: 95% confidence intervals. In order to compute OR (95% CI) the following alleles were used as reference: in correspondence with the allele of risk: rs2073617 T, rs3134070 T, and rs3134069 C.
